# Impact of the calibration bougie diametre during laparoscopic sleeve gastrectomy on the rate of postoperative staple-line leak (BOUST): study protocol for a multicentre randomized prospective trial

**DOI:** 10.1186/s13063-021-05734-3

**Published:** 2021-11-15

**Authors:** Martin Gaillard, Panagiotis Lainas, Hélène Agostini, Ibrahim Dagher, Hadrien Tranchart

**Affiliations:** 1 Department of Minimally Invasive Digestive Surgery, Hôpital Antoine Béclère, AP-HP, Université Paris-Saclay, 157 rue de la Porte de Trivaux, 92140 Clamart, France; 2 Faculté de Médecine Paris-Sud, Université Paris-Saclay, Orsay, France; 3 Department of Biostatistics and Clinical Research, Hôpital Bicêtre, AP-HP, Université Paris-Saclay, Le Kremlin Bicêtre, France

**Keywords:** Bariatric surgery, Obesity, Morbid, Postoperative complications, Gastrectomy, Surgical stapling, Calibration, Weight loss

## Abstract

**Background:**

Laparoscopic sleeve gastrectomy (LSG) has become an increasing bariatric procedure. The basic principle is to create a narrow stomach along the lesser curvature, using a calibration bougie as a template to perform a vertical partial gastrectomy, resecting the greater curvature and fundus of the stomach. The most common postoperative complication is gastric leak from the staple line, observed in approximately 3% of cases, which can result in long and incapacitating treatment. The diametre of the bougie used to calibrate the remnant stomach could impact the rate of postoperative gastric leak, a higher diametre being correlated with a lower risk of leak, without lowering long-term weight loss. This is the first randomized trial to compare the outcomes of LSG regarding the use of two different bougie diametres on postoperative gastric leak and mid-term weight loss.

**Methods:**

Bougie Sleeve Trial (BOUST) is a superiority single-blinded randomized national trial, involving 17 centres. Participants will be randomized into two groups. LSG will be performed using a 48-Fr diametre calibration bougie in the experimental group and a standard care (34 to 38-Fr diametre) calibration bougie in the control group. Both groups will take part in a 2-year postoperative follow-up to assess postoperative gastric leak rate and weight loss and quality of life evolution.

**Discussion:**

This study protocol will allow the investigators to determine if the use of a larger calibration bougie during LSG is associated with lower postoperative gastric leak occurrence without impairing mid-term weight loss and quality of life. The results of this trial will provide important data on patient safety and promote best practice for LSG procedures.

**Trial registration:**

ClinicalTrials.govNCT02937649. Registered on 18 October 2016

**Supplementary Information:**

The online version contains supplementary material available at 10.1186/s13063-021-05734-3.

## Administrative information

Note: the numbers in curly brackets in this protocol refer to SPIRIT checklist item numbers. The order of the items has been modified to group similar items (see http://www.equator-network.org/reporting-guidelines/spirit-2013-statement-defining-standard-protocol-items-for-clinical-trials/).

**Table:** 

Title {1}	Impact of the calibration bougie diametre during laparoscopic sleeve gastrectomy on the rate of postoperative staple-line leak (BOUST): study protocol for a multicentre randomized prospective trial
Trial registration {2a and 2b}.	ClinicalTrials.gov Identifier: NCT02937649
Protocol version {3}	Version N°4.0, 13 August 2020
Funding {4}	Programme Hospitalier de Recherche Clinique (PHRC) 2015 (Ministère de la Santé, France).
Author details {5a}	1. Department of Minimally Invasive Digestive Surgery, Hôpital Antoine Béclère, AP-HP, Université Paris-Saclay, Clamart, France. 2. Faculté de Médecine Paris-Sud, Université Paris-Saclay, Orsay, France. 3. Department of Biostatistics and Clinical Research, Hôpital Bicêtre, AP-HP, Université Paris-Saclay, Le Kremlin Bicêtre, France. * Correspondence: Dr. Hadrien Tranchart, MD, PhD, Department of Minimally Invasive Digestive Surgery, Hôpital Antoine Béclère, 157 rue de la Porte de Trivaux, 92140, Clamart, France. E-mail: hadrien.tranchart@aphp.fr.
Name and contact information for the trial sponsor {5b}	AP-HP, and by delegation: Clinical research and Innovation Delegation (DRCI) Hôpital Saint-Louis, 1, avenue Claude Vellefaux, 75010, Paris, France. DRCI-Siège project referent: Candy Estevez. Email : candy.estevez@aphp.fr
Role of sponsor {5c}	The sponsor is involved in overall study activities including study design, collection, management, analysis and interpretation of data, writing of the report, and decision to submit the report for publication.

## Introduction

### Background and rationale {6a}

Laparoscopic sleeve gastrectomy (LSG) has become an increasing bariatric procedure, mostly because of its relative simplicity and efficacy. Approximately 27,500 LSGs were performed in France in 2014 [1] and 125,000 in the USA in 2016 [2]. The basic principle is to create a narrow stomach along the lesser curvature, using a calibration bougie as a template to perform a vertical partial gastrectomy, resecting the greater curvature and fundus of the stomach. The most common complication is gastric leak resulting from staple-line leakage, observed in approximately 3% of cases [3]. Gastric leak is most often diagnosed within 5 days after surgery, but late presentations are described. Treatment of this complication is usually long and incapacitating, requiring surgical, radiological or endoscopic drainage and parenteral or enteral nutritional support. Controversy is ongoing about potential options that might reduce the risk of gastric leak, and among them the diametre of the bougie used to calibrate the remnant stomach’s volume. The issue is whether a sleeve gastrectomy with a smaller diametre is associated with greater weight loss at the expense of a higher rate of postoperative leak.

In a meta-analysis published in 2013 [3], the use of a bougie diametre larger than or equal to 40-French (Fr) and under 50-Fr was correlated with a decrease in the rate of leak, without lowering weight loss at 3 years. Most teams use a bougie diametre of less than 40-Fr [3], mainly 36-Fr [4], in order to reduce the risk of long-term weight regain. However, concerns of bariatric surgeons whether the use of a larger bougie could lead to weight regain [5] appear to be unfounded in the literature [3, 6]. Conversely, the use of a bougie larger than 45-Fr allows reduction of gastric leak rate to 0.92–1.7% [3, 6]. Indeed, no prospective randomized study was ever conducted to investigate the impact of the diametre of the calibration bougie on the rate of postoperative gastric leak. The main aim of this study is to show a reduction of postoperative gastric leak rate with the use of a 48-Fr diametre calibration bougie during LSG in comparison with the use of a smaller standard care bougie.

### Objectives {7}

This is a randomized trial comparing two strategies during the same surgical procedure, aiming to compare the outcomes of LSG according to the use of a standard care bougie diametre (34-, 36- or 38-Fr) or large (48-Fr) diametre bougie.

1. The primary objective is to compare the rate of postoperative gastric leak after usage of a 48-Fr diametre bougie *versus* a standard care diameter bougie during LSG during the first month of postoperative course.

2. Secondary objectives are to assess the impact of bougie diametre on (1) global postoperative morbidity, (2) short- and mid-term weight loss and (3) short- and mid-term quality of life.

### Trial design {8}

**Fig. 1:**
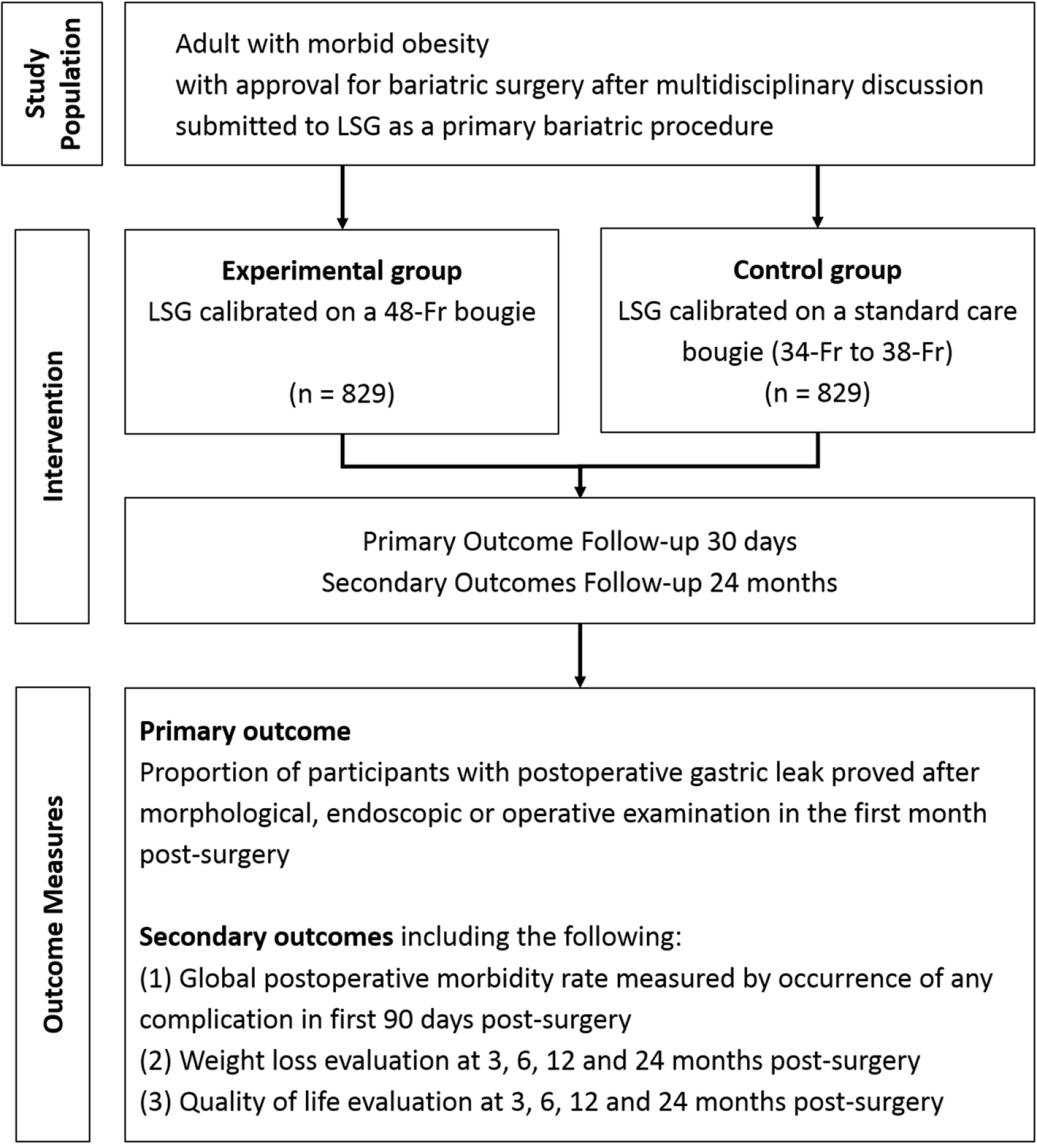
Outline of the trial. LSG, laparoscopic sleeve gastrectomy; Fr, French

BOUST is an investigator-initiated, pragmatic, prospective, multicentre, parallel-group, single-blind, randomized, active-controlled, superiority clinical trial. An outline of the trial is shown in Fig. 1. The experimental group is a 48-Fr group: participants will undergo LSG calibrated on a 48-Fr bougie. The control group is the standard care bougie group: participants will undergo LSG calibrated on a 34-Fr, 36-Fr or 38-Fr bougie according to the centre standard care. Participants will be distributed between groups in a 1:1 ratio according to stratified randomization. We used the Standard Protocol Items Recommendations for Interventional Trials (SPIRIT) Checklist to guide the reporting of our protocol [7].

## Methods: participants, interventions and outcomes

### Study setting {9}

A total of 1658 participants undergoing LSG will be recruited in 17 French centres (13 academic hospitals and 4 private hospitals). All centres are accredited by the French Health Agency for obesity management and bariatric surgery and perform more than 100 sleeve gastrectomy per year. Additional file 1 contains the list of the study sites. Local investigators are listed in the “Acknowledgements” section.

### Eligibility criteria {10}

#### Inclusion criteria

The population of the study involves all patients submitted to LSG, since staple-line leakage can occur in all patients. The inclusion criteria are:

1. Adult patient (age between 18 and 70 years) undergoing LSG as a primary bariatric procedure
2. Body mass index (BMI) superior to 40 kg/m^2^ or superior to 35 kg/m^2^ when associated with at least one comorbidity susceptible to improve after surgery (including arterial hypertension, obstructive sleep apnea syndrome and other severe respiratory disorders, severe metabolic disorders, particularly type 2 diabetes, incapacitating osteo-articular disorders, non-alcoholic steatohepatitis)
3. Decision for bariatric surgery approved after multidisciplinary discussion
4. Written informed consent

#### Exclusion criteria

The exclusion criteria are:

1. History of previous upper abdominal surgery (cholecystectomy excepted)
2. Score of the American Society of Anesthesiologists (ASA) superior to 3
3. Ongoing pregnancy or breast feeding
4. Oesophageal pathology or disorder (oesophageal varices, oesophageal diverticula, oesophageal tumours, oesophageal strictures)
5. Coagulation disorder
6. Known silicon hypersensitivity
7. Patients not covered by social security service or under legal guardianship and trusteeship

Previous upper abdominal surgery is associated with a higher risk of complicated postoperative course that would have confounded the trial [8–10]. Patients with ASA score > 3, ongoing pregnancy or breast feeding have been excluded for safety reasons. A systematic pregnancy test is routinely performed before bariatric surgery, and a negative result will be checked before enrolment. Patients with known silicon hypersensitivity (as 48-Fr calibration bougie contains medical silicon) and patients with oesophageal pathology or disorder (that could interfere with safe insertion and placement of the bougie) are also excluded for safety reasons. All surgical procedures will be included, whatever the age, gender, experience and position of the patient’s senior surgeon.

#### Drop-out criteria

The drop-out criteria are:

1. Consent withdrawal
2. Safety concerns
3. Selection criteria violations

Participants may exit the study for personal reason at any time. In this case, no new data will be collected since the date of withdrawal. Data collected prior to the date of withdrawal may still be used. In case of serious adverse reaction related to the usage of the experimental bougie, the investigator must notify the sponsor without delay. The participation will be discontinued but the participant will continue to be monitored for the study.

### Who will take informed consent? {26a}

Patients who are candidates for LSG at participating hospitals are invited to participate in the trial. Surgeons determine participant eligibility, discuss the trial and seek informed consent during routine bariatric preoperative assessments. All surgeons involved in obtaining consent and enrolling participants have received specific training in the trial and the requirements of Good Clinical Practice (GCP). They do not necessarily need to be investigators or research staff.

### Additional consent provisions for collection and use of participant data and biological specimens {26b}

Not applicable.

## Interventions

### Explanation for the choice of comparators {6b}

Usage of calibration bougie during sleeve gastrectomy has become an international standard [6], yet no recommendation regarding the bougie diametre has been proposed. Most teams use a bougie diametre of less than 40-Fr [3], mainly 36-Fr [4]. Meta-analysis and systematic review of observational studies have associated the use of a bougie diametre larger than or equal to 40-Fr with a decrease in the rate of leak [3, 11]. However, as with all observational studies, these findings may be affected by selection bias and residual confounding, making causal inferences problematic. No prospective randomized study has investigated the impact of the bougie diametre on the rate of postoperative gastric leak. Increasing the bougie diametre would not affect the surgical procedure, and no failure of introduction of a larger bougie has ever been reported [3]. Most teams preferring a large calibration bougie diametre use a 48-Fr bougie [6]. Hence, the diametre of the bougie in the experimental group was decided to be 48-Fr.

### Intervention description {11a}

Participants will be randomized to one of the two blinded bougie diametre groups:

1. Experimental group: LSG calibrated on a 48-Fr (16 mm) bougie
  or
2. Control group: LSG calibrated on a 34-Fr (11.3 mm), 36-Fr (12 mm) or 38-Fr (12.7 mm) bougie according to the centre standard care

After randomization, the anaesthesiologist will collect the corresponding blister containing the calibration bougie. During LSG, the calibration bougie is inserted through the mouth of the patient and placed in the stomach by the anaesthesiologist after gastric dissection. The surgeon uses the bougie to calibrate the remnant stomach during gastric section and stapling. The bougie is thereafter removed during the surgery by the anaesthesiologist. After removal, the bougie will be discarded according to routine practice in each centre. The use of both bougies is the same and instructions will be given to all anaesthesia departments.

**Fig. 2:**
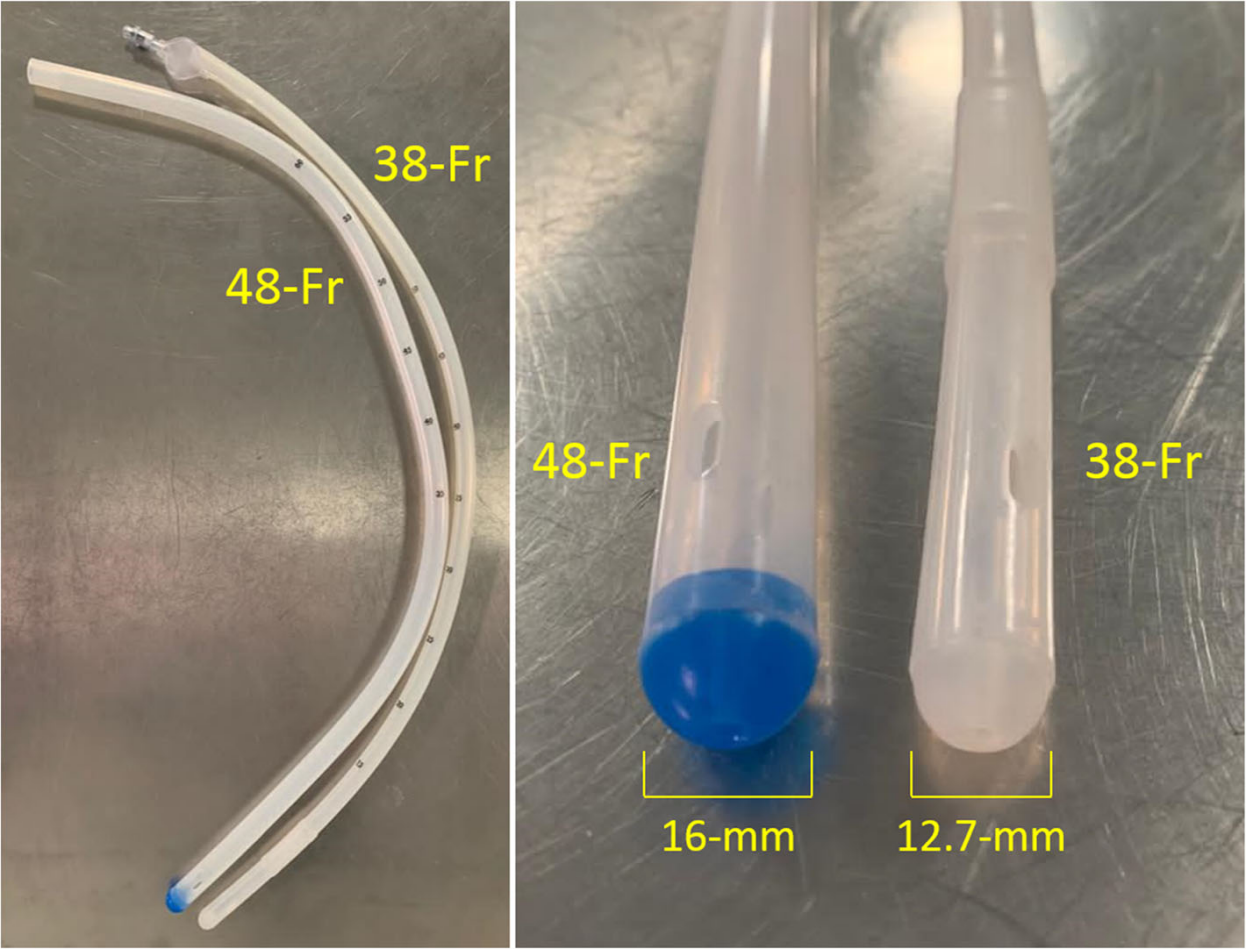
Experimental 48-Fr bougie and standard care 38-Fr bougie

Participants allocated in the experimental group will be assigned an individual blister containing the 48-Fr calibration bougie labelled with a unique study code. Experimental bougie is MID-TUBE orogastric calibration tube by Médical Innovation Développement S.A.S (ref. MID131). This is a CE-marked class IIa medical single-use device used in the field of gastroenterology. The device is 791 mm long, in medical silicon rubber, and contains no latex. The experimental bougie is provided by the sponsor to each centre’s pharmacy. Participants allocated in the control group will be assigned a standard care bougie (34-Fr, 36-Fr or 38-Fr) used in accordance with the manufacturer’s instructions. All standard care bougies are single-use devices, used in the indication of their CE marking, which allows the devices to be covered by the national insurance funds. Standard care bougies will not be provided by the sponsor, but they will be supplied by each centre’s pharmacy according to standard care and reimbursed by the French health social system. Experimental 48-Fr bougie and standard care 38-Fr bougie are presented in Fig. 2.

### Criteria for discontinuing or modifying allocated interventions {11b}

Intervention may be discontinued by the anesthesiologist at any time if the placement of the bougie is considered inadequate. In this case, the patient remains included in the study but will be treated according to the standard care procedure in each centre. The patient will be analysed in the experimental group according to the “intention-to-treat analysis”.

### Strategies to improve adherence to interventions {11c}

The intervention will be implemented entirely by local teams; thus, adherence to the intervention will be improved by three different methods. First, a preliminary face-to-face workshop will be held in each site, gathering site investigators and research staff to present the project in details, to tailor local resources including checklists and trial alert notices in medical records. Recommendations will be given during the preliminary workshop to standardize the surgical procedure, allowing decreasing the variability between centres. The standardized protocol is used as common practice in the coordinating investigator department, highly experimented in laparoscopic sleeve gastrectomy [12]. Second, a local project liaison will be the direct link between the local investigators and the study team, participating in the implementation of the intervention in each centre. The local project liaison will carry out regular follow-up visits in each centre to verify that the study is carried out in accordance with the current version of the protocol and all statutory and regulatory requirements. Finally, the study team will form a steering committee providing technical expertise and mentorship on project management.

### Relevant concomitant care permitted or prohibited during the trial {11d}

All participants receive usual postoperative management as per the local standard of care. This includes perioperative anaesthesia, surgical care, routine prophylactic measures, postoperative diet and management of complications. Systematic morphological imaging is not mandatory.

### Provisions for post-trial care {30}

Following the intervention, participants are followed up until 24 months after LSG. All postoperative care is provided by the centre where the LSG is performed as per routine practice. The sponsor will provide full compensation for any damages caused by the study to the study participants and their beneficiaries, unless the sponsor can prove that the harm is not the fault of the sponsor or any agent.

### Outcomes {12}

#### Primary outcome measure

The primary outcome is the occurrence of postoperative gastric leak. After LSG, gastric leak is known to occur within the first postoperative month [11]. Gastric leak is consistently a symptomatic complication, as no leak is assumed to stay unnoticed. To date, there is no consensus on the method for the diagnosis of gastric leak. Some teams use systematic morphological examination, and others prefer to rely on clinical and biological examination and proceed in case of suspicion of gastric leak either to immediate reintervention or to morphological investigation. No systematic examination for early detection of gastric leak has been proven to be reliable. In this study, diagnosis of postoperative gastric leak during the first month following the procedure must be fulfilled with either (1) morphologic examination (with contrast ingestion) such as abdominal CT scan or gastrointestinal swallow studies showing extravasation of the contrast material or abnormal perigastric pneumoperitoneum; (2) evidence of staple-line disruption by exit of blue dye during exploratory surgical reintervention or through the drainage during postoperative course; or (3) evidence of staple-line disruption by extravasation of contrast opacification during endoscopy. The method for the diagnosis of gastric leak does not depend on bougie diametre. The occurrence of postoperative gastric leak is notified by the surgeon during the first 3 months following the procedure.

#### Secondary outcome measures

Secondary outcomes are the following:

1. Global postoperative morbidity, defined as any complication occurring 90 days following the procedure; the severity of postoperative morbidity will be assessed according to the Dindo-Clavien classification of surgical complications [13] (Table 1).
2. Short- and mid-term weight loss
3. Short- and mid-term quality of life

**Table 1:** The Clavien-Dindo classification of surgical complications

Grades	Definition
Grade I	Any deviation from the normal postoperative course without the need for pharmacological treatment or surgical, endoscopic and radiological interventions Allowed therapeutic regimens are drugs as antiemetics, antipyretics, analgetics, diuretics and electrolytes and physiotherapy. This grade also includes wound infections opened at the bedside.
Grade II	Requiring pharmacological treatment with drugs other than such allowed for grade I complications Blood transfusions and total parenteral nutrition are also included.
Grade III	Requiring surgical, endoscopic or radiological intervention
Grade IIIa	Intervention not under general anaesthesia
Grade IIIb	Intervention under general anaesthesia
Grade IV	Life-threatening complication (including CNS complications)* requiring IC/ICU management
Grade IVa	Single organ dysfunction (including dialysis)
Grade IVb	Multiorgan dysfunction
Grade V	Death of a patient

*Brain haemorrhage, ischemic stroke, subarrachnoidal bleeding, but excluding transient ischemic attacks (TIA); *IC* intermediate care, *ICU* intensive care unit

In order to determine weight loss evolution, the initial weight will be measured and recorded by the surgeon the day before the procedure. Assessment of short-term weight loss will be performed at 3 and 6 months after the procedure, and assessment of mid-term weight loss will be performed at 1 and 2 years after the procedure by the surgeon using the percentage of excess weight loss and percentage of total weight loss. Percentage excess weight loss (%EWL) is calculated using the formula: %EWL=weight loss (kg)/excess weight (kg). Excess weight is based on the patient’s ideal weight with a BMI of 25 kg/m^2^. Percentage total weight loss (%TWL) is calculated using the formula: %TWL=weight loss (kg)/initial weight (kg).

Quality of life related to health will be assessed at 3 months, 6 months, 1 year and 2 years after the procedure with the French validated version [14] of the GIQLI (Gastrointestinal Quality of Life Index) questionnaire [15] (Additional file 2).

### Participant timeline {13}

As recommended by French Health Authorities, all patient candidates for bariatric surgery take part in a preoperative follow-up of at least 6 months before undergoing bariatric surgery [16]. During this preoperative course, patients will receive an explanation on the study protocol and will be given an information form. The enrolment phase takes place between 2 months and no later than 1 day before surgery. Participants are followed up in the trial from randomization until 24 months after LSG. Follow-up visits are scheduled as standard care after bariatric surgery. Data collection occurs at baseline and at follow-up visits at 3 months ± 1 month, 6 months ± 1 month, 1 year ± 3 months and 2 years ± 3 months after surgery. The participant timeline is presented in Table 2.

**Table 2:** Trial schedule of enrolment, intervention and assessments, as recommended by Standard Protocol Items: Recommendations for Interventional Trials (SPIRIT). *M* months, *D* day, *LSG* laparoscopic sleeve gastrectomy, *GIQLI* Gastrointestinal Quality of Life Index

Study period						
	Enrolment	Allocation	Follow-up			
Timepoint	M-2 to D-1	D0	M3	M6	M12	M24
Enrolment						
Eligibility screen	X					
Informed consent	X					
Baseline characteristics	X					
Allocation		X				
Interventions						
LSG with experimental bougie		X				
LSG with standard care bougie		X				
Assessments						
Postoperative gastric leak			X			
Global morbidity			X			
Weight	X		X	X	X	X
GIQLI questionnaire			X	X	X	X

### Sample size {14}

The sample size for the trial (1658 participants) is based on a comparison between two independent groups of the proportions of participants experiencing the primary outcome measure of postoperative gastric leak. We made the hypothesis of a difference of postoperative fistula rates of 3% with a standard care calibration bougie and 1% with a 48-Fr calibration bougie. Considering a bilateral alpha risk of 5% and a power of 80%, we would need to include 1576 patients (788 in each group). A 5% rate of participants lost to follow-up is estimated; therefore, 1658 patients will be included (829 in each group).

### Recruitment {15}

BOUST is a national multicentre research project conducted in 17 centres, each centre performing more than 100 LSG per year. Considering a total number of subjects to enrol of 1658 and an inclusion period of 24 months, the number of subjects to enrol by centre by month is 4 to 5.

Strategies have been implemented to achieve optimal participant enrolment. The trial was designed as a pragmatic protocol with large eligibility criteria. Recruitment of participants is achieved by the surgeon who supervises the preoperative assessments prior to LSG, performs the surgical procedure and fulfils follow-up. An Internet-based secure platform is used for simple and rapid enrolment, randomization and data collection. Trial processes are aligned to usual clinical care after bariatric surgery. Telephone support is provided to local investigators with the ability to access assistance for enrolment and randomization from an investigator of the coordinating centre.

## Assignment of interventions: allocation

### Sequence generation {16a}

Participants are randomized in a 1:1 ratio to either the experimental group (participants will undergo LSG calibrated on a 48-Fr bougie) or the control group (participants will undergo LSG calibrated on a standard care bougie). In order to reduce the imbalance between participants in each group, stratification will be carried out regarding known risk factors for postoperative gastric leak: gender and super-obesity (defined as BMI > 50 kg/m^2^). In each centre, the control group matches with the standard care procedure and the experimental group an identical procedure except for the usage of a 48-Fr bougie. Thereby, stratification will also be carried out regarding centres to reduce the imbalance between participants in each group. The block size would not be known by the investigator as the sample size is sufficient to ensure a correct repartition between the different strata. A potential imbalance at the end of randomization will be taken into account in the statistical analysis. No stratification regarding the modalities of the laparoscopic approach (conventional or single-port laparoscopy) has been defined as the modalities of the laparoscopic approach are consistent with the centre.

### Concealment mechanism {16b}

Randomization is performed via a secure server dedicated to electronic management of clinical research (CleanWEB®, Telemedicine Technologies, Boulogne-Billancourt, France). The code for generating group allocation was developed by a statistician independent to the project.

### Implementation {16c}

Participants are enrolled by local investigators between 2 months and no later than 1 day before surgery. Inclusion is completed using an Internet-based platform. Randomization is performed on the day of surgery by local investigators via a secure server. After allocation, the assigned calibration bougie is used during LSG.

## Assignment of interventions: blinding

### Who will be blinded {17a}

This is a single-blind randomized clinical trial, as study participants are blinded. After randomization, the anaesthesiologist will collect the corresponding blister containing the calibration bougie and will insert the bougie through the mouth in the stomach before the gastric section. As much as possible, the anaesthesiologist will try to prevent the surgeon from identifying which bougie is used. However, since the aspect and conditioning of experimental bougies are different than those of standard care bougies, it is difficult to ensure the blinding of the surgeon before, during and after the surgical procedure. Nonetheless, the blinding will be maintained for the participant and biostatisticians throughout the study and analyses.

### Procedure for unblinding if needed {17b}

Procedures for unblinding are not necessary since the use of standard care or 48-Fr bougie does not change intraoperative or postoperative management.

## Data collection and management

### Plans for assessment and collection of outcomes {18a}

Data is collected using an electronic case report form (CRF) via CleanWEB®. Trial processes are embedded within the CRF to improve trial efficiency. Clinical data are collected by study staff from the participant’s medical record. Information on health-related quality of life is collected by participant completion of the GIQLI questionnaire.

### Plans to promote participant retention and complete follow-up {18b}

As routine care after bariatric surgery, patients accept to take part in a multidisciplinary follow-up programme. Follow-up visits are timed to coincide with routine outpatient clinic appointments undertaken as part of standard care after LSG. The study staff collects the data required for each visit from medical records. In case of discontinuation or deviation from intervention protocol, the CRF will list the various reasons why the participant has discontinued the study. If a participant discontinues the study, this will in no way affect their usual care for his condition. If a participant exits the study prematurely or withdraws consent, any data collected prior to the date of premature exit may still be used. If a participant is lost to follow-up, the investigator must make every effort to reconnect with the participant, at least to determine whether the participant is alive or dead. Since there is a low chance of not evaluable subject, if a subject is not evaluable, his identification number will not be reallocated and he will not be replaced.

### Data management {19}

All data required for the protocol are collected using a CRF via CleanWEB® at each time point of the follow-up. Non-identifying data will be entered by a clinical research technician. Sources of collected data are medical records and appointment records. Source documents will be kept by each centre in case of medical reports. Documents specific to the trial will be archived by the investigator and the sponsor for 15 years following the end of the study in accordance with national guidelines. Data entry will be carried out on electronic media via a Web browser. Secured login and password will be attributed by a data manager. The CRF’s programming will limit invalid data as much as possible by restricting the possible values to type during the online completion of CRF. Each missing data item must be coded.

### Confidentiality {27}

Any information that may identify a participant will be excluded from the data presented in the public arena. All study-related information is stored securely in each study site. CRF are identified by a coded identification only and stored on secure servers. The electronic data collected for the study is used in accordance with French (“Informatique et Libertés” law governing data protection) and European (General Data Protection Regulation) regulations. This research is governed by the CNIL (French Data Protection Agency). Study staff, as well as the investigators themselves, are bound by professional secrecy (in accordance with the conditions set out in Articles 226-13 and 226-14 of the Code Pénal).

### Plans for collection, laboratory evaluation and storage of biological specimens for genetic or molecular analysis in this trial/future use {33}

No biological specimens are collected in this study.

## Statistical methods

### Statistical methods for primary and secondary outcomes {20a}

Analyses will be by intention to treat. The impact of the bougie calibration diametre, 48-Fr versus 34- to 38-Fr, on the primary outcome of postoperative gastric leak rate during the first postoperative month will be analysed using a logistic regression model, with group, and stratification factors (centre, gender, super-obesity). The primary comparison will be a two-sided test at the 5% significance level. The impact of the bougie calibration diametre on secondary outcomes will be analysed using statistical methods appropriate for the type of outcome. Logistic regression will be used in a multivariate model to identify factors independently associated with morbidity. The normal distribution will be tested using the Shapiro-Wilk test. Should this be significant at the 5% level, a non-parametric approach (Mann-Whitney test) or variable transformation will be used.

### Interim analyses {21b}

An interim analysis will be fulfilled when half of the patients (829) have been included to possibly prematurely stop the trial, based on the primary outcome, according to O’Brien Fleming Alpha Spending function method. The research will be stopped if the interim analysis demonstrates the efficacy of one of the arms being treated or due to futility. This analysis will be performed to ensure that there is no major imbalance of efficacy or tolerance (mortality and serious adverse effects surgical postoperative complications) between the two groups. The steering committee will discuss the discontinuation of the study in case of imbalance between postoperative gastric leak rate in each arm (if postoperative gastric leak rate is 5% higher in the experimental group compared to the control group).

### Methods for additional analyses (e.g. subgroup analyses) {20b}

Not applicable.

### Methods in analysis to handle protocol non-adherence and any statistical methods to handle missing data {20c}

Analysis will be performed on the intention-to-treat population. Methods used to handle missing data will be described fully in the study report.

### Plans to give access to the full protocol, participant-level data and statistical code {31c}

N/A

## Oversight and monitoring

### Composition of the coordinating centre and trial steering committee {5d}

The trial steering committee is chaired by the lead principal investigator for the study and includes the scientific director, the executive manager of the clinical research unit, a surgeon, an anaesthesiologist, a statistician and project management staff. The committee is responsible for drafting and amending the protocol and other key trial documents; monitoring recruitment, data entry completion and quality; and drafting and approving the statistical analysis plan and other trial publications. The steering committee will meet every 6 months during the recruitment period and at least once a year during the follow-up period. The committee will be consulted by the sponsor when the results of the intermediate analysis will be available. Actions will be proposed if necessary and the sponsor will communicate its final decision.

### Composition of the data monitoring committee, its role and reporting structure {21a}

This trial compares standard care *versus* experimental bougie during LSG. The bougie diametre does not change intraoperative or postoperative management nor the method for diagnosis of gastric leak. Besides, the experimental device is classified IIa, CE-marked, and has already been used for sleeve gastrectomy in previous essays. In view of the minimal risks arising from this research, it was not considered necessary to establish an independent data monitoring oversight committee. Nonetheless, the investigator will notify the sponsor without delay in case of serious adverse events. The sponsor will report to the French national safety agency without delay any serious adverse event possibly related to the usage of the experimental bougie.

### Adverse event reporting and harms {22}

Serious adverse events (SAEs) that are considered related to the study are monitored and reported without delay. SAEs related to the implementation action of the experimental medical device include perforation of the oesophagus or the stomach during bougie introduction, stapling and/or section of the bougie with the stapling device, impossibility to remove the bougie and staple-line opening during bougie removal. Study-specific reportable adverse events, including death, or resulting in a life-threatening situation or significant disability, or requiring inpatient hospitalization or prolongation of existing hospitalization are also being monitored during the trial.

All SAEs and reportable adverse events must be recorded in the CRF. The severity of adverse events is assessed according to the Dindo-Clavien classification of surgical complications [13].

### Frequency and plans for auditing trial conduct {23}

GCP compliance monitoring is being conducted by the sponsor. Risk-based monitoring is in place for the study. All data, documents and reports may be subject to regulatory audits and inspections.

### Plans for communicating important protocol amendments to relevant parties (e.g. trial participants, ethical committees) {25}

All protocol amendments will be approved by responsible independent ethics committees and local sites prior to implementation. Protocol amendment of August 5, 2020, has required re-consent from 1 participant of the study.

### Dissemination plans {31a}

Trial findings will be disseminated at national and international scientific meetings, by publication in a scientific journal. Authorship for all trial publications will be based on criteria formulated by the International Committee of Medical Journal Editors, available at www.icmje.org.

## Discussion

Surgical strategies that reduce the risk of gastric leak after LSG have the potential to improve postoperative outcomes and reduce costs. This multicentre, prospective, randomized, single-blind, pragmatic trial has been designed to determine whether the diametre of the bougie used to calibrate the remnant stomach during LSG is important, specifically whether the use of a 48-Fr bougie decreases the rate of postoperative gastric leak. Given that the use of a calibration bougie is an international standard, evidence for the superiority of a 48-Fr bougie over a smaller diametre standard care bougie from this trial would provide strong justification for a change in surgical practices. Considering the large sample size and the scarcely restrictive eligibility criteria, a generalizability of results is expected to other populations undergoing LSG. A finding of no significant difference between the groups for the primary and key secondary outcomes would imply that any benefit with the use of a 48-Fr bougie does not exist, or is not clinically important for the majority of patients undergoing LSG. Regardless, BOUST will provide data on the safety of larger diametre calibration bougie, particularly in relation to intraoperative complications.

In line with the pragmatic study design, and to promote the recruitment of a large number of participants and the generalizability of the trial results, eligibility criteria were designed to enrol a large range of patients. It is estimated that more than 90% of patient candidates for LSG are eligible for this study. Trial processes have been designed to be in line with routine clinical care at participating centres, enabling surgeons to easily enrol and monitor their patients without the presence of research staff. All data collected, apart from the quality of life questionnaires, are already routinely collected. No additional laboratory tests or procedures are required for the trial.

In the protocol for BOUST originally approved by ethics committee, before the enrolment started (protocol version 1.0, 10 June 2016), the operating surgeon was kept blinded of the diametre of the calibration bougie inserted by the anaesthesiologist. Considering that the surgeon would become aware of which bougie is used, either by direct visualization of the bougie in the operating room or its shape in the empty stomach, the study protocol was amended in 2019 (protocol version 2.0, 5 August 2019) to change the double-blind design to a patient-blind design. This change aligns with standard practice, since the bougie diametre is routinely determined by the surgical team and the patient is generally unaware of the diametre of the bougie used.

There are some limitations that need to be considered. By opting for a pragmatic design, BOUST does not mandate any specific methods to use during the perioperative period or during surgery that could impact the risk of postoperative gastric leak, including the diametre of the standard care bougie. Several other surgical methods have been advocated (distance between the staple-line and the pylorus, buttressing material or suture of the staple-line, staple heights) but none has demonstrated a decrease of gastric leak rate [3]. However, these technical modalities of LSG are standardized in each study site and stratified randomization by centre will be carried out. Mid-term weight loss evolution is a key secondary outcome of the study. A concern of bariatric surgeons and patients is that the use of a larger bougie could lead to long-term (> 5 or 10 years after LSG) weight regain. Although BOUST was designed to analyse the effect of the bougie diametre on the primary outcome, this trial will be, to our knowledge, the largest randomized study to report the outcomes of the calibration bougie diameter on mid-term weight loss. Longer-term follow-up studies may help determine if a larger calibrated sleeve is associated with weight regain.

In conclusion, BOUST is an ongoing, randomized, controlled, pragmatic trial that will provide the most definitive comparative data to date on the risk of gastric leak after LSG using 48-Fr vs. 34–38-Fr calibration bougie. If the hypothesis that 48-Fr bougie is superior to 34–38-Fr bougie is proven, this will provide an important strategy to improve LSG outcomes, which can be rapidly implemented into clinical practice.

## Trial status

This article refers to protocol version 4 dated 13 August 2020. The recruitment has begun on 8 October 2020. The anticipated recruitment end date is 7 October 2022.

## Supplementary Information

**Additional file 1.** List of active study sites.

**Additional file 2.** French version of the Gastrointestinal Quality of Life Index (GIQLI) questionnaire.

**Additional file 3.** Information sheet and consent form.

**Additional file 4.** Ethical approval document (French version) and English translation.

## Abbreviations

ASA: American Society of Anesthesiologists
BMI: Body mass index
CNIL: Commission nationale de l'informatique et des libertés
CRF: Case report form
DSMB: Data and Safety Monitoring Board
EWL: Excess weight loss
Fr: French
GCP: Good Clinical Practice
GIQLI: Gastrointestinal Quality of Life
LSG: Laparoscopic sleeve gastrectomy
SAE: Serious adverse event
TWL: Total weight loss
