# Supplementary material for: Impact of the calibration bougie diametre during laparoscopic sleeve gastrectomy on the rate of postoperative staple-line leak (BOUST): study protocol for a multicentre randomized prospective trial
Source: Trials. 2021 Nov 15;22:806. doi: 10.1186/s13063-021-05734-3 (PMC8591884; doi:10.1186/s13063-021-05734-3)
Supplement: Supplementary file 1 — Additional file 1. List of active study sites. [file 13063_2021_5734_MOESM1_ESM.docx]

**Additional File 1**

List of active study sites

| Hôpital Antoine Béclère, Clamart, Île-de-France |
| --- |
| Hôpital Bichat-Claude Bernard, Paris, Île-de-France |
| CHU Grenoble Alpes, Grenoble, Auvergne-Rhône-Alpes |
| CHU Reims, Reims, Grand-Est |
| GH Diaconesses Croix Saint-Simon, Paris, Île-de-France |
| CHU Amiens Picardie, Amiens, Hauts-de-France |
| Clinique Notre Dame, Vire, Normandie |
| CHU Limoges, Limoges, Nouvelle-Aquitaine |
| CHU Caen Normandie, Normandie |
| CHI Poissy Saint Germain, Poissy, Île-de-France |
| CHI Créteil, Créteil, Île-de-France |
| Clinique de l’Yvette, Longjumeau, Île-de-France |
| Hôpital Ambroise Paré, Boulogne-Billancourt, Île-de-France |
| CHU Nancy, Nancy, Grand-Est |
| Clinique Mutualiste Chirurgicale, Saint Etienne, Auvergne-Rhône-Alpes |
| CH Saint Denis, Saint Denis, Île-de-France |
| Hôpital Louis Mourier, Colombes, Île-de-France |
