# Supplementary material for: Impact of the calibration bougie diametre during laparoscopic sleeve gastrectomy on the rate of postoperative staple-line leak (BOUST): study protocol for a multicentre randomized prospective trial
Source: Trials. 2021 Nov 15;22:806. doi: 10.1186/s13063-021-05734-3 (PMC8591884; doi:10.1186/s13063-021-05734-3)
Supplement: Supplementary file 2 — Additional file 2. French version of the Gastrointestinal Quality of Life Index (GIQLI) questionnaire. [file 13063_2021_5734_MOESM2_ESM.docx]

**Additional File 2**

French version of the Gastrointestinal Quality of Life Index (GIQLI) questionnaire.

1. Durant les 15 derniers jours, vous avez eu mal au ventre :

toujours (0) ; la plupart du temps (1) ; quelques fois (2) ; rarement (3) ; jamais (4)

2. Durant les 15 derniers jours, vous avez eu la sensation d’avoir l’estomac gonflé :

toujours (0) ; la plupart du temps (1) ; quelques fois (2) ; rarement (3) ; jamais (4)

3. Durant les 15 derniers jours, vous avez eu la sensation d’avoir beaucoup de gaz dans le ventre :

toujours (0) ; la plupart du temps (1) ; quelques fois (2) ; rarement (3) ; jamais (4)

4. Durant les 15 derniers jours, vous avez été gêné(e) par l’émission de « vents » :

toujours (0) ; la plupart du temps (1) ; quelques fois (2) ; rarement (3) ; jamais (4)

5. Durant les 15 derniers jours, vous avez été gêné(e) par des éructations ou des renvois :

toujours (0) ; la plupart du temps (1) ; quelques fois (2) ; rarement (3) ; jamais (4)

6. Dans les 15 jours qui ont précédé, vous avez été gêné(e) par des bruits de « glouglou » dans le ventre :

toujours (0) ; la plupart du temps (1) ; quelques fois (2) ; rarement (3) ; jamais (4)

7. Durant les 15 derniers jours, vous avez été gêné(e) par des selles fréquentes :

toujours (0) ; la plupart du temps (1) ; quelques fois (2) ; rarement (3) ; jamais (4)

8. Durant les 15 derniers jours, vous avez mangé avec plaisir et appétit :

toujours (0) ; la plupart du temps (1) ; quelques fois (2) ; rarement (3) ; jamais (4)

9. A cause de votre intervention, vous êtes obligé(e) de supprimer certains aliments :

de façon très importante (0) ; de façon importante (1) ; un peu (2) ; un tout petit peu (3) ; pas du tout (4)

10. Durant les 15 derniers jours, vous avez été capable de surmonter les problèmes quotidiens :

extrêmement mal (0) ; mal (1) ; modérément (2) ; bien (3) ; extrêmement bien (4)

11. Durant les 15 derniers jours, combien de fois votre intervention vous a-t-elle rendu(e) triste :

toujours (0) ; la plupart du temps (1) ; quelques fois (2) ; rarement (3) ; jamais (4)

12. Durant les 15 derniers jours, combien de fois avez-vous été anxieux(se) à cause de votre intervention :

toujours (0) ; la plupart du temps (1) ; quelques fois (2) ; rarement (3) ; jamais (4)

13. Durant les 15 derniers jours, combien de fois avez-vous ressenti la joie de vivre :

toujours (0) ; la plupart du temps (1) ; quelques fois (2) ; rarement (3) ; jamais (4)

14. Durant les 15 derniers jours, combien de fois avez-vous été frustré(e) à cause de votre intervention :

toujours (0) ; la plupart du temps (1) ; quelques fois (2) ; rarement (3) ; jamais (4)

15. Durant les 15 derniers jours, combien de fois vous êtes-vous senti(e) fatigué(e) :

toujours (0) ; la plupart du temps (1) ; quelques fois (2) ; rarement (3) ; jamais (4)

16. Durant les 15 derniers jours, combien de fois avez-vous été souffrant :

toujours (0) ; la plupart du temps (1) ; quelques fois (2) ; rarement (3) ; jamais (4)

17. Durant la dernière semaine, vous êtes-vous réveillé(e) pendant la nuit :

toutes les nuits (0) ; 5 ou 6 nuits (1) ; 3 ou 4 nuits (2) ; 1 ou 2 nuits (3) ; jamais (4)

18. Depuis que vous avez été opéré(e), avez-vous été chagriné(e) par les modifications de votre apparence :

pour une grande part (0) ; modérément (1) ; un peu (2) ; un tout petit peu (3) ; pas du tout (4)

19. A quel degré est-ce que l’intervention a réduit votre condition physique en général :

énormément (0) ; beaucoup (1) ; un peu (2) ; un tout petit peu (3) ; pas du tout (4)

20. A cause de votre intervention, vous avez perdu de votre endurance :

pour une grande part (0) ; modérément (1) ; un peu (2) ; un tout petit peu (3) ; pas du tout (4)

21. De par votre intervention, vous estimez la perte de votre tonus :

majeure (0) ; modérée (1) ; minime (2) ; insignifiante (3) ; nulle, vous êtes en forme (4)

22. Durant les 15 derniers jours, combien de fois avez-vous été capable d’accomplir vos activités habituelles (travail, école, ménage, etc.) :

jamais (0) ; rarement (1) ; quelques fois (2) ; la plupart du temps (3) ; toujours (4)

23. Durant les 15 derniers jours, vous avez été capable de vaquer à vos loisirs habituels ou d’entreprendre de nouvelles activités :

jamais (0) ; rarement (1) ; quelques fois (2) ; la plupart du temps (3) ; toujours (4)

24. Durant les 15 derniers jours, avez-vous été incommodé(e) par le traitement médical :

énormément (0) ; beaucoup (1) ; un peu (2) ; un tout petit peu (3) ; pas du tout (4)

25. Dans quelle mesure votre intervention perturbe-t-elle vos relations avec les autres (famille ou amis) :

pour une très grande part (0) ; pour une part importante (1) ; un peu (2) ; un tout petit peu (3) ; pas du tout (4)

26. Dans quelle mesure votre intervention a-t-elle causé du tort à votre vie sexuelle :

pour une très grande part (0) ; pour une part importante (1) ; un peu (2) ; un tout petit peu (3) ; pas du tout (4)

27. Durant les 15 derniers jours, combien de fois avez-vous été incommodé(e) par des remontées de liquide ou d’aliments dans la bouche (régurgitations) :

toujours (0) ; la plupart du temps (1) ; quelques fois (2) ; rarement (3) ; jamais (4)

28. Durant les 15 derniers jours, vous êtes-vous senti(e) obligé(e) de diminuer la vitesse avec laquelle vous mangez :

toujours (0) ; la plupart du temps (1) ; quelques fois (2) ; rarement (3) ; jamais (4)

29. Durant les 15 derniers jours, vous avez eu des problèmes pour avaler :

toujours (0) ; la plupart du temps (1) ; quelques fois (2) ; rarement (3) ; jamais (4)

30. Durant les 15 derniers jours, vous avez ressenti le besoin urgent d’aller à la selle :

toujours (0) ; la plupart du temps (1) ; quelques fois (2) ; rarement (3) ; jamais (4)

31. Durant les 15 derniers jours, vous avez été incommodé(e) par de la diarrhée :

toujours (0) ; la plupart du temps (1) ; quelques fois (2) ; rarement (3) ; jamais (4)

32. Durant les 15 derniers jours, vous avez été incommodé(e) par une constipation :

toujours (0) ; la plupart du temps (1) ; quelques fois (2) ; rarement (3) ; jamais (4)

33. Durant les 15 derniers jours, vous avez été incommodé(e) par une nausée :

toujours (0) ; la plupart du temps (1) ; quelques fois (2) ; rarement (3) ; jamais (4)

34. Durant les 15 derniers jours, vous avez été inquiété(e) par la présence de sang dans les selles :

toujours (0) ; la plupart du temps (1) ; quelques fois (2) ; rarement (3) ; jamais (4)

35. Durant les 15 derniers jours, vous avez été incommodé(e) par une brûlure ou une acidité́ remontant dans la poitrine :

toujours (0) ; la plupart du temps (1) ; quelques fois (2) ; rarement (3) ; jamais (4)

36. Durant les 15 derniers jours, vous avez été incommodé(e) par une incontinence pour les selles :

toujours (0) ; la plupart du temps (1) ; quelques fois (2) ; rarement (3) ; jamais (4)
