# Supplementary material for: Impact of the calibration bougie diametre during laparoscopic sleeve gastrectomy on the rate of postoperative staple-line leak (BOUST): study protocol for a multicentre randomized prospective trial
Source: Trials. 2021 Nov 15;22:806. doi: 10.1186/s13063-021-05734-3 (PMC8591884; doi:10.1186/s13063-021-05734-3)
Supplement: Supplementary file 3 — Additional file 3. Information sheet and consent form. [file 13063_2021_5734_MOESM3_ESM.pdf]

Titre complet de recherche :
Etude prospective randomisée multicentrique évaluant l'effet du diamètre du tube de calibration utilisé au cours des sleeve gastrectomies laparoscopiques sur le taux de fistule postopératoire sur la ligne d'agrafage

Cette recherche est promue par l'Assistance Publique - Hôpitaux de Paris
Représentée par la Directrice de la Direction de la Recherche Clinique et de l'Innovation
1 avenue Claude Vellefaux
75010 Paris

NOTE D'INFORMATION

Madame, Monsieur,

Le Docteur/Professeur……………………………………. (nom, prénom), exerçant à l'hôpital ……………………………………………., vous propose de participer à une recherche concernant votre maladie.
Il est important de lire attentivement cette note avant de prendre votre décision ; n'hésitez pas à lui demander des explications.
Si vous décidez de participer à cette recherche, un consentement écrit vous sera demandé.

1) Quel est le but de cette recherche?

Vous êtes pris en charge pour votre obésité et allez être opéré(e) d'une sleeve gastrectomie laparoscopique, appelée communément sleeve. Cette opération consiste à retirer les deux tiers de l'estomac, pour le transformer en un tube. Le volume de l'estomac restant est calibré à l'aide d'une sonde. Cette sonde est insérée par la bouche et positionnée dans l'estomac au début de l'intervention, et retirée à la fin. La complication la plus fréquente de cette procédure est l'apparition d'une fuite au niveau de la ligne d'agrafage qui permet de refermer la partie de l'estomac restant. Cette complication peut conduire à un traitement long et invalidant.

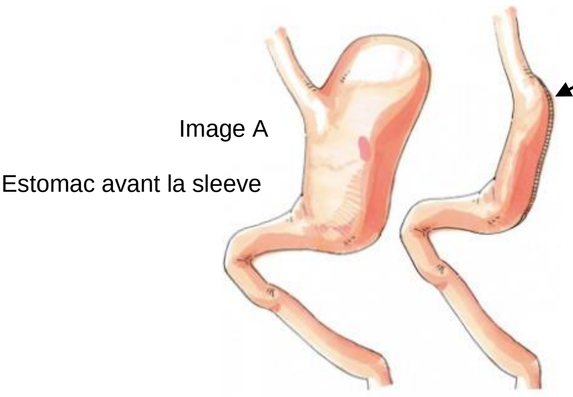

Le but de cette étude est de comparer les sondes utilisées dans le cadre du soin (sondes entre 11,3 mm et 12,7mm de diamètre) avec les sondes de diamètre 16 mm. Nous souhaitons démontrer que l'utilisation de la sonde de 16 mm diminue le taux de fuites de la ligne d'agrafage après la sleeve qui est actuellement d'environ 3%..
Pour répondre à cette question , il est prévu d'inclure environ 1658 personnes qui seront opérées d'une sleeve, dans différents hôpitaux et cliniques sur le territoire français.

Ce document est la propriété du DRCD / APHP. Toute reproduction est formellement interdite.

2) En quoi consiste la recherche ?

Dans la recherche proposée, nous allons évaluer si l'utilisation de la sonde de 16 mm permet de diminuer le taux de fuite après la sleeve en comparaison avec la sonde actuellement utilisée par chaque service participant lors d'une sleeve.
Les bilans préopératoires sont réalisés dans le cadre du soin ainsi que la procédure utilisée pour réaliser la sleeve.. Les sondes utilisées seront les sondes œsophagiennes habituelles utilisées par les services pour les sondes du soin et les sondes achetées auprès de la société MID pour les sondes de 16mm. L'anesthésiste sera chargé de l'introduction de la sonde.
Si vous acceptez de participer à l'étude, le diamètre de la sonde que vous recevrez sera tiré au sort ; vous ne serez pas informé de la sonde assignée. La méthode pour diagnostiquer les fuites n'est pas dépendante du diamètre de la sonde.

3) Quel est le calendrier de la recherche ?

Votre participation sera au maximum de 27 mois après votre intervention. Le suivi avant et après l'intervention sera le même que celui réalisé lors de la prise en charge habituelle de votre obésité. Dans un premier temps, vous serez informé(e) des objectifs et des risques de cette étude au cours d'une consultation avec votre chirurgien. Dans un deuxième temps, votre consentement sera récupéré et vous devrez compléter un questionnaire concernant votre qualité de vie. Ces deux phases se dérouleront avant l'intervention et pourront être fusionnées.
Le tirage au sort se fera par l'anesthésiste le jour de l'opération, la sonde sera placée puis retirée au cours de l'intervention.

Après l'intervention, comme prévu pour la prise en charge des patients opérés d'une sleeve, vous aurez 4 consultations postopératoires obligatoires à 3, 6 mois, 1 et 2 ans après l'intervention. En supplément des évaluations prévues lors de ces consultations, il vous sera demandé pour la recherche de compléter un questionnaire sur la qualité de vie.

4) Quels sont les bénéfices liés à votre participation ?

Vous contribuerez à améliorer les connaissances portant sur la prévention des complications liées à la sleeve.

5) Quels sont les traitements autorisés et non autorisés ?

Tout traitement pris antérieurement à la recherche, ou nécessaire au cours de votre hospitalisation et votre suivi est autorisé.

6) Quels sont les risques et les contraintes prévisibles ajoutés par la recherche?

Une sonde sera placée dans l'estomac lors de la réalisation de la sleeve gastrectomie. Cette procédure est indépendante de la recherche.
Les risques liés à l'utilisation de la sonde sont connus :

- Ouverture de l'œsophage ou de l'estomac,
- Agrafage ou section de la sonde par la pince utilisée pour couper et agrafer l'estomac,
- Problèmes lors du retrait de la sonde de l'estomac (saignement, plaie muqueuse).

L'objectif de cette étude est de trouver un moyen de réduire le risque de fuite sur la ligne d'agrafage, aujourd'hui évalué à environ 3%.
La recherche implique seulement que le diamètre de cette sonde soit différent de celui qui est utilisé habituellement en cas de randomisation dans le groupe « sonde de diamètre de 16 mm ».
Cette recherche devrait permettre d'identifier le diamètre de la sonde qui diminuerait le risque de fuite.

Si vous acceptez de participer, vous devrez respecter les points suivants :

- Conserver sur vous en permanence la carte patient qui vous a été remise, indiquant que vous participez à cette recherche. Cette carte inclut le nom et le numéro de téléphone de votre médecin.
- Venir aux rendez-vous. En cas d'impossibilité, nous vous remercions de contacter votre chirurgien le plus rapidement possible.
- Informer votre chirurgien de l'utilisation de tout médicament ainsi que de tout événement survenant pendant la recherche (hospitalisation, grossesse,…).
- Ne pas prendre part à un autre projet de recherche sans l'accord de votre chirurgien, ceci pour vous protéger de tout accident possible.
- Etre affilié(e) à un régime de sécurité sociale ou être bénéficiaire d'un tel régime.

Ce document est la propriété du DRCD / APHP. Toute reproduction est formellement interdite.

7) Quelles sont les éventuelles alternatives médicales?

A la fin de la recherche ou en cas d'arrêt prématuré de la recherche quelle qu'en soit la cause, un suivi médical adapté vous sera proposé selon les pratiques de votre centre.

En cas de non réalisation de la procédure de la recherche, le suivi initialement prévu pour la recherche sera poursuivi. Ce cas peut se présenter par exemple si l'anesthésiste ne parvient pas à introduire la sonde tirée au sort.

8) Quelles sont les modalités de prise en charge médicale à la fin de votre participation?

En fin de recherche ou en cas d'arrêt prématuré de la recherche (sur votre décision ou celle de l'investigateur), votre prise en charge ne sera pas modifiée et vous continuerez vos soins comme initialement prévus.

9) Si vous participez, comment vont être traitées les données recueillies pour la recherche ?

Dans le cadre de la recherche à laquelle il vous est proposé de participer, un traitement de vos données personnelles va être mis en œuvre par l'AP-HP, promoteur de la recherche, et responsable de traitement, pour permettre d'en analyser les résultats. Ce traitement est nécessaire à la réalisation de la recherche qui répond à la mission d'intérêt public dont est investie l'AP-HP en tant qu'établissement public de santé hospitalo-universitaire.

A cette fin, les données médicales vous concernant et les données relatives à votre qualité de vie « digestive » seront transmises au Promoteur ou aux personnes ou partenaires agissant pour son compte en France. Ces données seront identifiées par un numéro d'enregistrement. Ces données pourront également, dans des conditions assurant leur confidentialité, être transmises aux autorités de santé françaises.

Les données médicales vous concernant pouvant documenter un dossier auprès des autorités compétentes portant sur le Dispositif Médical évalué dans cette recherche, pourront être transmises à un industriel afin qu'un plus grand nombre de patients puissent bénéficier des résultats de la recherche. Cette transmission sera faite dans les conditions assurant leur confidentialité.

Vos données pourront être utilisées pour des recherches ultérieures ou des analyses complémentaires à la présente recherche en collaboration avec des partenaires privés ou publics, en France ou à l'étranger, dans des conditions assurant leur confidentialité et le même niveau de protection que la législation européenne.
Vous pouvez vous opposer à tout moment à l'utilisation ultérieure de vos données auprès du médecin qui vous suit dans le cadre de cette recherche.

Vos données ne seront conservées que pour une durée strictement nécessaire et proportionnée à la finalité de la recherche. Elles seront conservées dans les systèmes d'information du responsable de traitement, jusqu'à deux ans après la dernière publication des résultats de la recherche.
Vos données seront ensuite archivées selon la réglementation en vigueur.

Le fichier informatique utilisé pour cette recherche est mis en œuvre conformément à la règlementation française (loi Informatique et Libertés modifiée) et européenne (au Règlement Général sur la Protection des Données -RGPD), vous disposez d'un droit d'accès, de rectification, de limitation et d'opposition au traitement des données couvertes par le secret professionnel utilisées dans le cadre de cette recherche. Ces droits s'exercent auprès du médecin en charge de la recherche qui seul connaît votre identité (identifié en première page du présent document).

Si vous décidez d'arrêter de participer à la recherche, les données recueillies précédemment à cet arrêt seront utilisées conformément à la réglementation, et exclusivement pour les objectifs de cette recherche. En effet, leur effacement serait susceptible de compromettre la validité des résultats de la recherche. Dans ce cas, vos données ne seront absolument pas utilisées ultérieurement ou pour une autre recherche.

En cas de difficultés dans l'exercice de vos droits, vous pouvez saisir le Délégué à la Protection des données de l'AP-HP à l'adresse suivante : protection.donnees.dsi@aphp.fr, qui pourra notamment vous expliquer les voies de recours dont vous disposez auprès de la CNIL. Vous pouvez également exercer votre droit à réclamation directement auprès de la CNIL par Internet : www.cnil.fr ou par voie postale : 3 place Fontenoy TSA 8715 PARIS CEDEX.

Ce document est la propriété du DRCD / APHP. Toute reproduction est formellement interdite.

10) Comment cette recherche est-elle encadrée ?

L'AP-HP a pris toutes les mesures pour mener cette recherche conformément aux dispositions du Code de la Santé Publique applicables aux recherches impliquant la personne humaine.

L'AP-HP a souscrit une assurance (0100518814033-160040-10998) garantissant sa responsabilité civile et celle de tout intervenant auprès de la compagnie HDI–GLOBAL SE par l'intermédiaire de BIOMEDICINSURE dont l'adresse est Parc d'Innovation Bretagne Sud C.P.142 56038 Vannes Cedex.

L'AP-HP a obtenu l'avis favorable du Comité de Protection des Personnes pour cette recherche (*CPP* Ile de France VII), le 12/10/2016 et une autorisation de l'Agence Nationale de Sécurité du Médicament et des produits de santé (ANSM) le 28/10/2016.

11) Quels sont vos droits ?

Votre participation à cette recherche est entièrement libre et volontaire. Votre décision n'entraînera aucun préjudice sur la qualité des soins et des traitements que vous êtes en droit d'attendre.

Avant d'accepter de participer à cette recherche, vous bénéficierez d'un examen médical adapté, dont les résultats vous seront communiqués.

Vous pourrez tout au long de la recherche demander des explications sur le déroulement de la recherche au médecin qui vous suit.

Vous pouvez vous retirer à tout moment de la recherche sans justification, sans conséquence sur la suite de votre traitement ni la qualité des soins qui vous seront fournis et sans conséquence sur la relation avec votre médecin. A l'issue de ce retrait, vous pourrez être suivi par la même équipe médicale. Dans ce cas, les données collectées jusqu'au retrait seront utilisées pour l'analyse des résultats de la recherche.

Votre dossier médical restera confidentiel et ne pourra être consulté que sous la responsabilité du médecin s'occupant de votre traitement ainsi que par les autorités de santé et par des personnes dûment mandatées par l'AP-HP pour la recherche et soumises au secret professionnel.

A l'issue de la recherche et après analyse des données relatives à cette recherche, vous pourrez être informé(e) des résultats globaux par l'intermédiaire du médecin qui vous suit dans le cadre de cette recherche.
Vous pouvez également accéder directement ou par l'intermédiaire d'un médecin de votre choix à l'ensemble de vos données médicales en application des dispositions de l'article L 1111-7 du Code de la Santé Publique.

Après avoir lu toutes ces informations, discuté tous les aspects avec votre médecin et après avoir bénéficié d'un temps de réflexion suffisant, si vous acceptez de participer à la recherche, vous devrez signer et dater le formulaire de consentement éclairé se trouvant à la fin de ce document.

Ce document est la propriété du DRCD / APHP. Toute reproduction est formellement interdite.

FORMULAIRE DE CONSENTEMENT

Je soussigné(e), Mme, M. *[rayer les mentions inutiles]* (*nom, prénom*)…………………………………………………………..
accepte librement de participer à la recherche intitulée
« **Etude prospective randomisée multicentrique évaluant l'effet du diamètre du tube de calibration utilisé au cours des sleeve gastrectomies laparoscopiques sur le taux de fistule postopératoire sur la ligne d'agrafage** »
organisée par l'Assistance Publique - Hôpitaux de Paris et qui m'est proposée par le Docteur (*nom, prénom, téléphone*)……………………………………………………………………………….…, médecin dans cette recherche.

- J'ai pris connaissance de la note d'information version 3.1 du 05/10/2020 (4 pages) m'expliquant l'objectif de cette recherche, la façon dont elle va être réalisée et ce que ma participation va impliquer,
- je conserverai un exemplaire de la note d'information et du consentement,
- j'ai reçu des réponses adaptées à toutes mes questions,
- j'ai disposé d'un temps suffisant pour prendre ma décision,
- j'ai compris que ma participation est libre et que je pourrai interrompre ma participation à tout moment, sans encourir la moindre responsabilité et préjudice pour la qualité des soins qui me seront prodigués.
- j'ai été informé que les données recueillies dans le cadre de la recherche peuvent être réutilisées pour des recherches ultérieures, et que je pouvais m'y opposer à tout moment,
- je suis conscient(e) que ma participation pourra aussi être interrompue par le médecin si besoin, il m'en expliquera les raisons,
- avant de participer à cette recherche, j'ai bénéficié d'un examen médical adapté à la recherche, dont les résultats m'ont été communiqués,
- j'ai compris que pour pouvoir participer à cette recherche je dois être affilié(e) à un régime de sécurité sociale ou bénéficiaire d'un tel régime. Je confirme que c'est le cas,
- j'ai bien été informé(e) que ma participation à cette recherche durera maximum 27 mois après l'intervention et que cela implique que je ne pourrai pas envisager de participer à une autre recherche avant la fin de ma participation à celle-ci, sans en informer le médecin qui me suit pour la recherche,
- mon consentement ne décharge en rien le médecin qui me suit dans le cadre de la recherche ni l'AP-HP de l'ensemble de leurs responsabilités et je conserve tous mes droits garantis par la loi.

**Signature de la personne participant à la recherche**		**Signature du médecin**	
Nom Prénom :		Nom Prénom :	
Date :	Signature :	Date :	Signature :

Ce document est à réaliser en 3 exemplaires, un exemplaire doit être conservé *15 ans* par l'investigateur, le deuxième transmis à l'AP-HP sous enveloppe scellée à la fin de la recherche et le troisième remis à la personne donnant son consentement.

Ce document est la propriété du DRCD / APHP. Toute reproduction est formellement interdite.
