# Supplementary material for: Impact of the calibration bougie diametre during laparoscopic sleeve gastrectomy on the rate of postoperative staple-line leak (BOUST): study protocol for a multicentre randomized prospective trial
Source: Trials. 2021 Nov 15;22:806. doi: 10.1186/s13063-021-05734-3 (PMC8591884; doi:10.1186/s13063-021-05734-3)
Supplement: Supplementary file 4 — Additional file 4. Ethical approval document (French version) and English translation. [file 13063_2021_5734_MOESM4_ESM.pdf]

Hôpital de Bicêtre – 78 rue du général Leclerc – 94275 Le Kremlin Bicêtre Cedex
Présidente : Anne-Marie TABURET – Secrétaire : Brigitte PILATE

Projet de Recherche N° : ***16-029*** Kremlin Bicêtre le 6 novembre 2020

Le Comité a été saisi le 17 août 2020
D'une modification substantielle par Madame ESTEVEZ du DRCD – Hôpital Saint Louis – 1 avenue Claude Vellefaux – 75475 PARIS CEDEX 10 pour le compte du promoteur AP-HP – 3 avenue Victoria – 75004 PARIS concernant le projet de recherche qui avait obtenu un avis favorable du C.P.P. le 18 octobre 2016, le 15 mars 2017 et le 15 novembre 2019 et intitulé :
« ESSAI PROSPECTIF RANDOMISE MULTICENTRIQUE EVALUANT L'EFFET DU DIAMETRE DE LA BOUGIE DE CALIBRATION UTILISEE AU COURS DES SLEEVE GASTRECTOMIES SUR LE TAUX DE FISTULE POST-OPERATOIRE SUR LA LIGNE D'AGRAFAGE»
Référence du promoteur : N° : P150933 – BOUST - ID-RCB : 2016-A00406-45)

dont le coordonnateur national est le Docteur TRANCHART – Service de Chirurgie Digestive – Hôpital Antoine Béclère – 157 rue de la Porte de Trivaux – 92141 CLAMART CEDEX

Le comité a examiné la modification substantielle n° 3 datée du 17 août 2020 aux séances du 16 septembre 2020 et du 14 octobre 2020 et portant sur la prorogation de l'étude de 12 mois, la mise à jour de la liste des investigateurs, la nouvelle actualisation de la note d'information et du formulaire de consentement (NIFC) au regard du RGPD et l'ajout d'un addendum à la NIFC pour les patients inclus avant la réception de l'avis du comité avec note d'information et formulaire de consentement version 3.1 du 05/10/2020, addendum version 1.1 du 05/08/2020 à la NIFC version 2.0 du 05/08/2019, addenda au protocole version 4.0 du 13/08/2020 en suivi de modifications, avec CV des nouveaux investigateurs : centre 008 (Limoges), Centre 009 Caen), Centres 013 et 513 (Ambroise Paré, Boulogne), centres 017 et 517 (Louis Mourier, Colombes), 505 (Diaconesses), 514 (CHU Nancy) et tableau de suivi des modifications du 17/08/2020

Membres présents lors de la délibération de votre protocole
Premier Collège :
- Recherche biomédicale : Monsieur P. de BOISSIEU, épidémiologiste (T), Monsieur M. BOTTLAENDER (T), Monsieur F. HIRSCH (T), Madame C. HILL (S), Monsieur G. P. de FILIPPO, pédiatre (T), Monsieur R. de BEAUREPAIRE (S) et Madame K. BOURDIC (S)
- Pharmacien : Madame A. M. TABURET (T)

Deuxième collège :
- Personne qualifiée en éthique : Monsieur P. CASAURANG (T)
- Juriste : Madame F. BOISSY (T) et Madame LAFOY (T)
- Associations agréées : Madame A. LABBE (T) et Monsieur C. COTTET (T) et Monsieur G. MARDUEL (S)

Le Comité :
- considérant que l'amendement en référence ne modifie ni le respect méthodologique, ni la protection des personnes participant à l'étude

a adopté la délibération suivante :

AVIS FAVORABLE sans restriction

pour François HIRSCH
Président de la séance plénière du 14 octobre 2020

ENGLISH TRANSLATION

Commitee for the Protection of Persons Ile-de-France VII

Bicetre Hospital – 78, rue du général Leclerc – 94275 Le Kremlin Bicêtre Cedex

President: Anne-Marie TABURET – Secretary: Brigitte PILATE

Research Project number: 16-029 Kremlin Bicêtre, November 6th 2020

The Commitee has been notified on August 17th 2020 of a substantial modification by Mrs. ESTEVEZ from DRCD (Clinical Research and Innovation Division) – Hôpital Saint Louis – 1, avenue Claude Vellefaux – 75475 PARIS CEDEX 10 on behalf of sponsor AP-HP (Assitance Publique Hôpitaux de Paris) – 3 avenue Victoria – 75004 PARIS concerning research project which received a favorable opinion from the CPP (Committee for the Protection of Persons) on October 18th 2016, March 15th 2017 and November 15th 2019 and entitled "**MULTICENTER RANDOMIZED PROSPECTIVE TRIAL ASSESSING THE IMPACT OF THE CALIBRATION BOUGIE DIAMETRE DURING LAPAROSCOPIC SLEEVE GASTRECTOMY ON THE RATE OF POSTOPERATIVE STAPLE-LINE LEAK**"

Sponsor reference number: P150933 – BOUST – ID-RCB 2016-A00406-45)

of which the national coordinator is Dr. TRANCHART – Department of Digestive Surgery – Antoine Beclere Hospital – 157 rue de la Porte de Trivaux – 92141 CLAMART CEDEX.

The Committee has considered the substantial modification number 3 dated August 17th 2020 during meetings on September 16th 2020 and October 14th 2020 on the extension of 12 months of the study period, the update of the list of the investigators, the new update of the information sheet and consent form (NIFC) in the light of Data Protection General Regulation (RGPD) and an addendum to the NIFC concerning patients included before reception of the opinion of the Committee with the information sheet and consent form version 3.1 of 05/10/2020, addendum version 1.1 of 05/08/2020 of the NIFC version 2.0 of 05/08/2019, addendum of the protocol version 4.0 of 13/08/2020 after

modifications, with curriculum vitae of new investigators: centre 008 (Limoges), Centre 009 Caen), Centres 013 et 513 (Ambroise Paré, Boulogne), centres 017 et 517 (Louis Mourier, Colombes), 505 (Diaconesses), 514 (CHU Nancy) and table of modifications of 17/08/2020.

Attending members during the discussion of your protocol

First Bureau:

- Biomedical research: Mr. P. de BOISSIEU, epidemiologist (T), Mr. M. BOTTLAENDER (T), Mr. F. HIRSCH (T), Mrs. C. HILL (S), Mr. G. P. de FILIPPO, paediatrician (T), Mr. R. de BEAUREPAIRE (S) and Mrs. K. BOURDIC (S)

- Pharmacist: Mrs. A. M. TABURET (T)

Second Bureau:

- Ethics expert: Mr. P. CASAURANG (T)

- Jurist: Mrs. F. BOISSY (T) and Mrs. LAFOY (T)

- Authorized associations: Mrs. A. LABBE (T) and Mr. C. COTTET (T) and Mr. G. MARDUEL (S)

The Committee:

- in view of the fact that the amendment above does not modify neither the methodological respect, nor the protection of the study participants, adopted the following decision:

FAVORABLE OPINION without restriction

for François HIRSCH, Chairman of the plenary meeting of October 14th 2020
